# Hydrolytic Degradation and Bioactivity of Electrospun PCL-Mg-NPs Fibrous Mats

**DOI:** 10.3390/molecules28031001

**Published:** 2023-01-19

**Authors:** Valentina Salaris, Daniel López, José Maria Kenny, Laura Peponi

**Affiliations:** 1 Instituto de Ciencia y Tecnología de Polímeros (ICTP-CSIC), C/Juan de la Cierva 3, 28006 Madrid, Spain; 2 Civil and Environmental Engineering Department and UDR INSTM, STM Group, University of Perugia, Strada di Pentima 4, 05100 Terni, Italy

**Keywords:** biodegradable polymers, biocomposites, electrospinning, PCL, Mg-based nanoparticles

## Abstract

In this work, the in vitro degradation behavior of nanofibers was investigated in phosphate buffer solution (PBS) and simulated body fluid (SBF) to study their degradation behavior, as well as their bioactivity. The degradation was studied at different immersion times in order to evaluate how the presence of Mg-based nanoparticles can affect the degradation in terms of morphology, crystallinity, degradation rate and pH changes, and finally to evaluate the bioactivity of PCL-based electrospun nanofibers. We found that the degradation of the materials takes more than 3 months; however, the presence of nanoparticles seems to have an accelerating effect on the degradation of the electrospun nanofibers based on PCL. In fact, a reduction in diameter of almost 50% was observed with the highest content of both types of nanoparticles and an increase in crystallinity after 296 days of immersion in PBS. Moreover, the carbonyl index was calculated from an FTIR analysis, and a reduction of 20–30% was observed due to the degradation effect. Additionally, the bioactivity of PCL-based electrospun nanofibers was studied and the formation of crystals on the nanofibers surface was detected, except for neat electrospun PCL related to the formation of NaCl and apatites, depending on the amount and type of nanoparticles. The presence of apatites was confirmed by an XRD analysis and FT-IR analysis observing the characteristic peaks; furthermore, the EDX analysis demonstrated the formation of apatites than can be reconducted to the presence of HA when 20 wt% of nanoparticles is added to the PCL electrospun fibers.

## 1. Introduction

Electrospinning is a low-cost and versatile technique for the production of polymeric fibers at the micro- and nano-level through the application of an electric field on a polymer solution or melt [1]. The morphology of the electrospun fibers can be controlled by varying different parameters among them, including those related to the equipment, such as applied voltage, flow rate and tip-to-collector distance; those related to the polymeric solution, such as concentration, solution conductivity, solvent, additives, viscosity, surface tension, etc., [2]; and those related to the surroundings, such as temperature and humidity.

However, this technique presents some issues that also limit its scalability at the industrial level: for example, the use of volatile solvents and low flow rates with a consequent low production rate of nanofibers, which can be overcome by the use of a multi-needle spinneret.

On the other hand, the applied electric field at each needle can affect the jets of other needles, and thus, the morphology of nanofibers. To overcome these challenges, one of the adopted approaches is the utilization of needleless electrospinning [3].

Furthermore, recent advances of the electrospinning technique allow fiber composition to be tailored using new electrospinning methods, such as blend electrospinning, emulsion electrospinning, coaxial electrospinning, triaxial electrospinning and parallel electrospinning, which permit the development of hybrid nanofibrous scaffolds comprising two or more polymeric matrices [4]. Moreover, one of the most recent developments of electrospinning involves the fabrication of advanced nanomaterials, which combine the properties related to nanofibers and additives for various applications; drug delivery is one such application, taking advantage of the high drug-loading capacity of fibrous materials [5]. In fact, the encapsulation of new functional ingredients in the electrospun nanofibers to endow them with new properties and functional performances is a key factor that still needs to be deeply studied. The functional ingredients can be small molecules, nanoparticles [5], drugs, herbal medicine, oil [6] and even cells [7]. Although nanofibrous hybrids are demonstrated to show the desired functional performances by taking advantage of the nanofiber’s unique properties, only very limited studies can be found about the influences of the additives on the properties of a filament-forming polymeric matrix. The introduction of reinforcements into the neat polymeric matrix enhances the physical, mechanical and biological properties of scaffolds due to their high surface area and high aspect ratio [8]. Various nanoparticles (NPs) have been added for their antibacterial and antitumor effect and appropriate osteoconductivity, such as zeolites and hydroxyapatite (HA) [9]. Other types of NPs, including graphene and its derivatives, such as graphene oxide (GO), are able to increase the ability of the scaffold to grow, proliferate and differentiate various types of cells onto the surface [8]. ZnO NPs have been successfully introduced into PLA electrospun nanofibers, improving the mechanical properties of the polymeric matrix and the bacteriostatic/bactericidal activity of the nanocomposite [10].

Among the various areas where electrospinning finds applications, the biomedical field is one of the most important, since the morphology, porosity and large surface area of the electrospun micro and nanofibers mimic the fibrillar features of a natural extracellular matrix [11]. In particular, the interconnected porous network makes these materials suitable as scaffolds for tissue engineering; in fact, these pores permit the loading of bioactive molecules and the transportation of nutrients during tissue regeneration [12]. Furthermore, the importance of using biodegradable and biocompatible materials is related to the advantage of their metabolization in the human body into biocompatible degradation products, thus, rendering a second surgery for implant removal unnecessary [13]. To date, over 100 types of biodegradable polymers, including collagen, chitosan, alginates, polyesters and polyurethanes, have been used in this process [14,15]. Aliphatic polyesters undergo degradation through hydrolysis of the ester bonds. Furthermore, the acidic byproducts have an autocatalytic effect on the degradation process [16]. Moreover, the degradation rate of aliphatic polyesters depends on various factors, such as structure, molecular weight, crystallinity, hydrophilicity, etc., and for this reason, poly(glycolic acid) (PGA) exhibits a faster degradation rate with respect to poly(lactic acid) (PLA) and poly(ε-caprolactone) (PCL), which present a slower degradation rate [17]. Moreover, taking advantage of its slow degradation rate, PCL, which is a biodegradable, biocompatible, hydrophobic aliphatic polyester, has been widely used in drug delivery and tissue engineering [18]. However, since one of the necessary requirements of a material used in tissue engineering is that the degradation rate of the implanted material coincides with the tissue regeneration rate [19], the slow degradation rate as well as the poor mechanical properties of PCL can be considered as its major limitations. In order to solve this, the introduction of nano-reinforcements is one of the approaches utilized to improve these properties [20]. Different nanocomposites based on PCL have been already obtained with organic and inorganic particles, such as cellulose nanocrystals (CNC), chitosan, graphene and hydroxyapatite (HA) [21,22]. Among NPs, magnesium (Mg)-based NPs have gained copious attention as novel materials for implants for their degradation, mechanical and physiological properties. Indeed, Mg is involved in different cellular processes, such as the synthesis of proteins, ATPase function and bone remodeling [23,24]. Furthermore, Mg-based NPs such as magnesium oxide (MgO) are reported to have non-toxic biological activity, biocompatibility, and are able to stimulate bone formation; one of the most important applications is as degradable material for tissue engineering and, in particular, hard tissues regeneration [23]. However, the rapid corrosion of Mg-based biomaterials in physiological conditions, which leads to the formation of hydrogen gas (H_2_), represents a great problem [25]. Therefore, with the aim of better controlling as well as tailoring the degradation rate of Mg-based biomaterials, the use of Mg-based NPs enveloped with biocompatible and biodegradable polymers is one of the adopted strategies [26]. Recently, Leones et al. [27] fabricated PLA-based electrospun fibers reinforced with MgO NPs at different concentrations and studied their mechanical and shape memory behavior. De Silva et al. [28] examined the physical properties of alginate-based nanofibrous scaffolds reinforced with MgO NPs. In our previous work, we carried out a comparative study between the thermal, mechanical and morphological properties of PCL-based electrospun nanofibers reinforced with two types of NPs: MgO and magnesium hydroxide Mg(OH)_2_ [29].

In this work, the in vitro degradation behavior of nanofibers was investigated in phosphate buffer solution (PBS) and simulated body fluid (SBF) to study their degradation behavior, as well as the bioactivity of these materials, at different immersion times, in order to study how the presence of NPs affects the degradation in terms of morphology, crystallinity, degradation rate and pH changes, and finally to evaluate the bioactivity of PCL-based electrospun nanofibers, named ePCLs.

## 2. Results

First, the different reinforced ePCL fibers were obtain and characterized, as reported elsewhere [21]. We reported that both NPs were added at 0.5, 1, 5, 10 and 20 wt% with respect to the polymeric matrix, and electrospun fibers with an average diameter in the range of nanometers were obtained, with high crystallinity and thermal stability. Furthermore, the mechanical properties were studied and produced an interesting result. We found that MgO and Mg(OH)_2_ NPs are able to improve the mechanical response of electrospun PCL fibers, in particular the formulation with Mg(OH)_2_.

**Figure 1:**
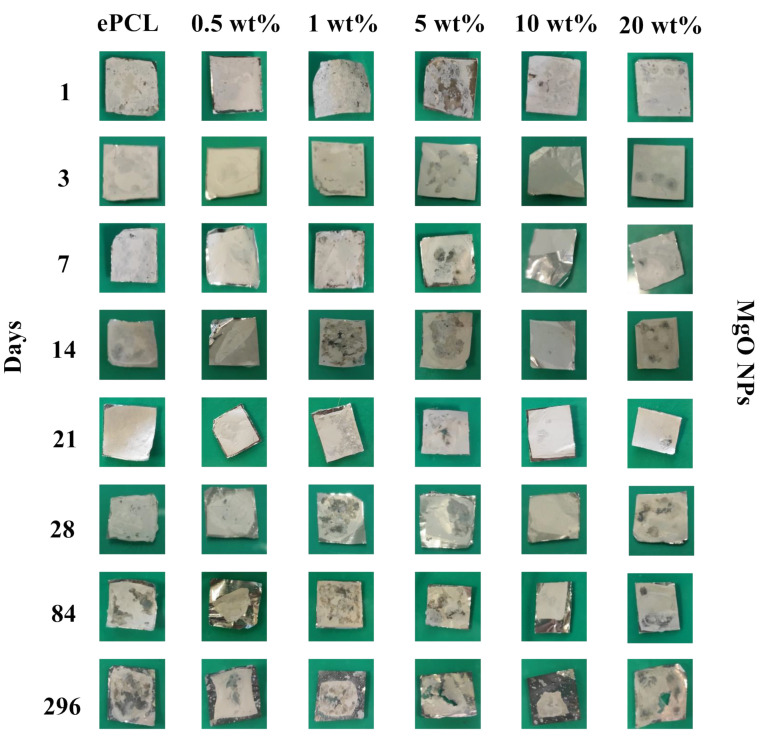
Photos of ePCL and ePCL + MgO NPs at different concentrations and different immersion times.

**Figure 2:**
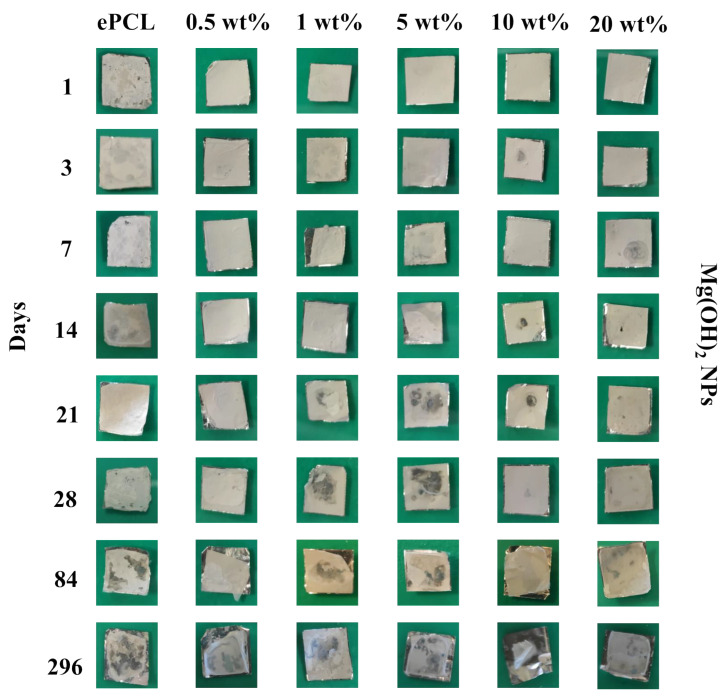
Photos of ePCL and ePCL + Mg(OH)_2_ NPs at different concentrations and different immersion times.

Therefore, once characterized by the different systems, we studied the in vitro degradation at different times, such as 1, 3, 7, 14, 21, 28, 84 and 296 days, which were called T1, T3, T7, T14, T21, T28, T84 and T296, respectively. Photos of each sample at each extraction time were taken in order to study the effect of the degradation from a macroscopic point of view, as reported in Figure 1 and Figure 2 for MgO and Mg(OH)_2_-based electrospun PCL fibers, respectively. The degradation process took almost one year, according to the slow degradation rate of PCL compared to other polymers [2,30].

The morphology of neat ePCL fibers did not show particular differences until T84. It can be seen from the T84 samples that the neat electrospun polymer started showing changes in terms of color and the disintegration of the material due to its degradation. In the case of ePCL with MgO NPs (Figure 1), this process was observed at T7, except for ePCL + MgO 5 wt%, which started on the first day of degradation. Moreover, from T84, besides the color changes and the disintegration of the material, the samples in all concentrations of NPs showed a reduction in size and demonstrated breaking. In the formulation with Mg(OH)_2_ NPs, no particular variations were observed until T21; some of the materials started showing disintegration at T28 but more evident differences were noticed at T84, and the reduction in size only occurred on the last day of the experiment.

From the macroscopical point of view, the ePCL nanofibers reinforced with NPs presented faster degradation with respect to the neat polymeric matrix—in particular, the formulation with MgO NPs.

**Figure 3:**
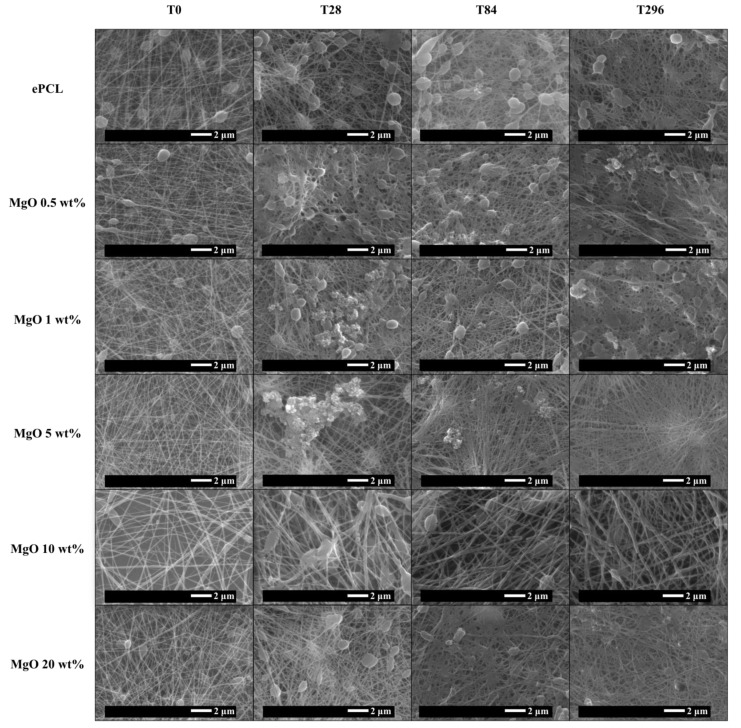
SEM images of ePCL and ePCL + MgO NPs at T0, T28, T84 and T296 in PBS.

**Figure 4:**
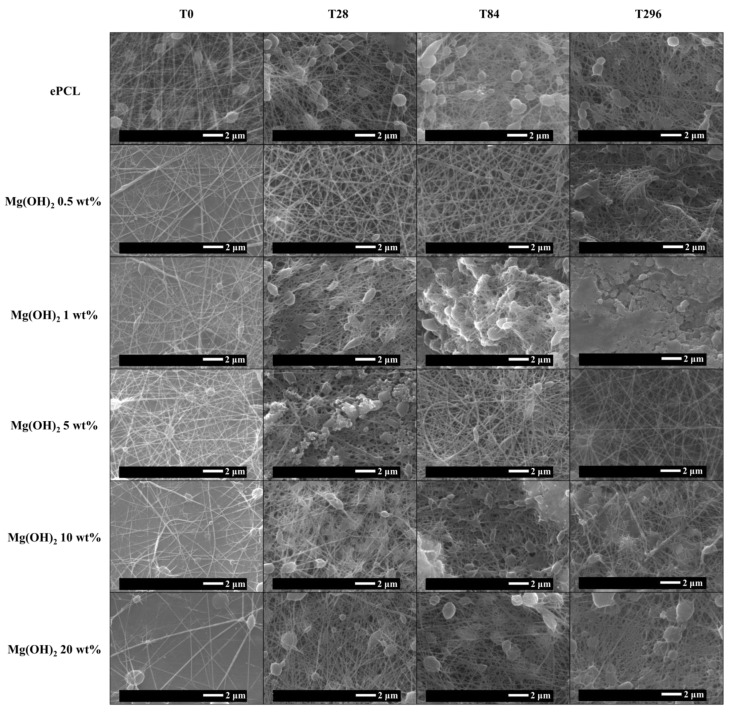
SEM images of ePCL and ePCL with Mg(OH)_2_ at T0, T28 T84 and T296 in PBS.

With the purpose of studying how degradation affects the electrospun nanofibers, it is important to evaluate not only the visual appearance of the samples, but also the microscopical changes, as well as the effects of the immersion on PBS in the morphology of the nanofibers. Therefore, the morphology of the fibers after 14, 21, 28, 84 and after 296 days of immersion in PBS was studied and compared to the fibers at time 0 of degradation, reported in Figure 3 and Figure 4 for the different systems based on MgO and Mg(OH)_2_, respectively, where SEM images at T0, T28, T84 and T296 are reported to show the morphological changes caused by the degradation in the different electrospun systems. We decided to only show these degradation times, as we considered them to be more significative.

**Figure 5:**
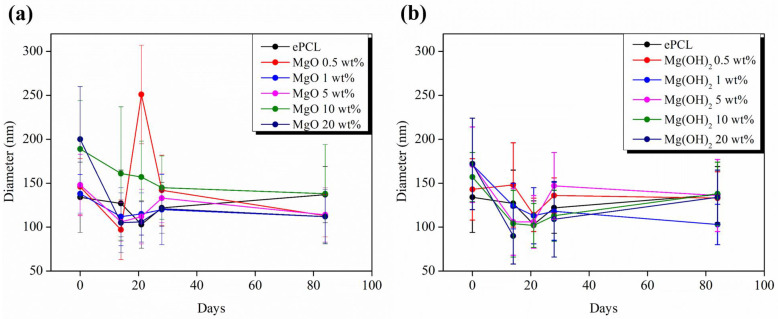
Variation of the electrospun fibers diameters at different days of degradation in PBS for: (**a**) ePCL reinforced with MgO NPs, (**b**) ePCL reinforced with Mg(OH)_2_ NPs.

Figure 3 and Figure 4 compare the nanofibers at different immersion times in order to better evaluate the morphological variations of the materials compared to the original electrospun nanofibers at T0. ePCL at T0 presents some beads, probably due to the concentration used, at 10 wt% in chloroform and a DFM ratio of 4:1. However, this concentration is optimized in order to disperse a high number of nanoparticles, such as 20 wt%, with respect to the polymeric matrix. However, it can be clearly observed that the degradation process affected the morphology of the electrospun nanofibers after 28 days; the surface became irregular, as caused by the surface hydrolytic erosion [31] that led to the rupture of the fibers at random points after 84 days; and the morphology was almost totally lost after 296 days [2]. Moreover the formation of minerals on the fiber surface after 28 days can be observed, which can be reconducted to a mineralization process [30], as was also confirmed by the XRD analysis. Furthermore, considering that we were working with electrospun polymeric fibers, the average diameter (Figure 5) was measured with ImageJ software and calculated as the average value of 30 random measurements for each sample, in order to study its variation with the diameter values of the electrospun fibers (efibers) obtained at T0.

For ePCL (black dots), no variations were observed in the diameter values during the first 84 days. ePCL presented an average diameter of 134 ± 40 nm at T0 and 137 ± 32 nm at T84. The value obtained at T0 is in accordance with those reported in the literature [21]. Nevertheless, the average efiber diameter slightly decreased from 134 ± 40 nm (T0) to 94 ± 31 nm after 296 days. This result confirms the slow degradation rate of PCL even in the form of electrospun fibers, with respect to other polymers. For example, PLA shows faster degradation, with a reduction in diameter after 7 days [30].

In the case of nano-reinforced electrospun nanofibers as well, the average diameter presented very similar values to the initial ones until T84. In fact, a reduction in the average fiber diameter was observed at T296, in particular with 20 wt% of NPs, which presented the initial highest diameter values. The diameter of ePCL + MgO 20 wt% decreased from 200 ± 60 nm (T0) to 134 ± 31 nm after 84 days and to 113 ± 28 nm after 296 days, with a reduction in diameter of 43%, and from 172 ± 52 nm (T0) to 134 ± 31 nm (T84) and to 93 ± 19 nm after 296 days for ePCL + Mg(OH)_2_ 20 wt%, with a reduction in diameter of 46%. Taking into account the obtained results, it seems that the presence of both NPs accelerates the degradation process, as reported with others NPs such as ZnO and HA [32,33].

**Figure 6:**
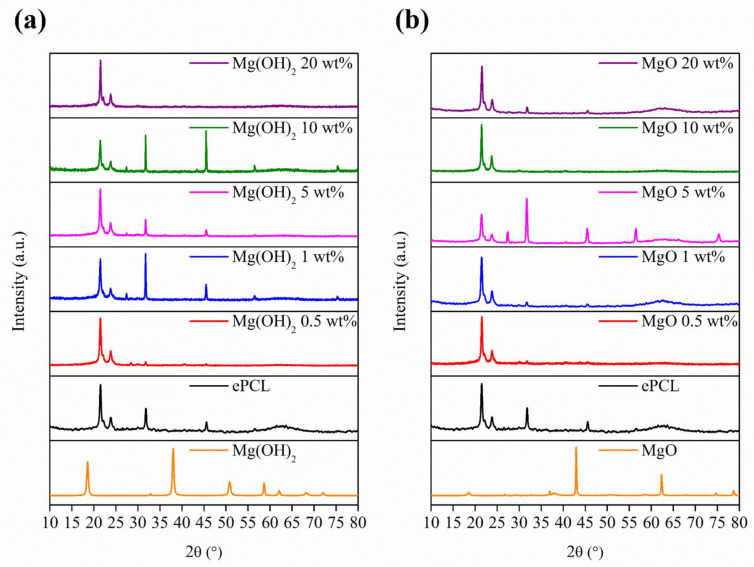
XRD analysis of ePCL reinforced with MgO NPs (**a**) and Mg(OH)_2_ NPs (**b**) at T296.

The results obtained by the XRD analysis are shown in Figure 6, where the diffraction patterns after 296 days are reported for ePCL reinforced with MgO and Mg(OH)_2_, respectively, in Figure 6a,b.

The diffraction peaks at 2θ = 21.5° and 23.9° are associated to the crystallographic planes [110] and [200] of PCL. No signals related to NPs were present after 296 days.

Furthermore, additional peaks were observed at 2θ = 27.4, 32, 45.5 and 56.5°, which are ascribed to the [200], [220] and [222] crystallographic planes of NaCl [34], and at 2θ = 28.5°, 40.5°, 53.8°, 66.4° and 75.32°, which are attributed to the face-centered cubic structure of KCl [35], corroborating the mineralization process observed by SEM.

**Scheme 1:**
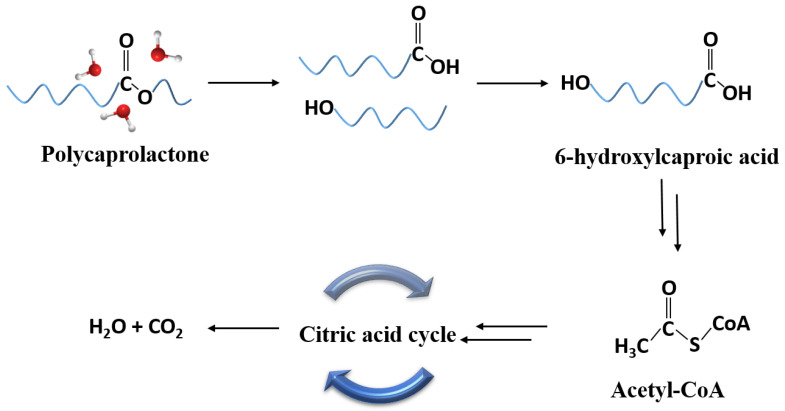
Schematic biodegradation mechanism and metabolic pathway of PCL.

Among various characterization techniques, the FTIR analysis permits the degradation process of ePCL and PCL-based electrospun nanofibers to be followed. In particular, the biodegradation of PCL involves the cleavage of the ester bond, leading to the formation of shorter polymeric chains and oligomers with carboxyl and hydroxyl end groups until the production of 6-hydroxycaproic acid, which is incorporated into the β-oxidation cycle to form 3-acetyl-CoA, which is finally metabolized in the citric acid cycle [19] (Scheme 1).

**Figure 7:**
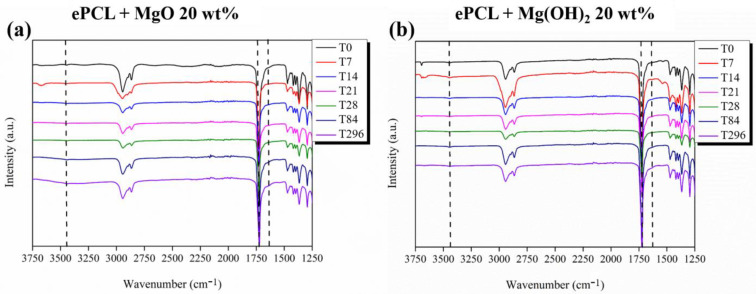
FTIR spectra of (**a**) ePCL + MgO 20 wt% and (**b**) ePCL + Mg(OH)_2_ 20 wt% at different extraction times.

Therefore, by monitoring the signals related to the ester, carboxylic and hydroxyl functional groups, it is possible to study the evolution of the degradation process. Thus, to better visualize the signal variation caused by degradation, only the FTIR spectra of ePCL + MgO 20 wt% and ePCL + Mg(OH)_2_ 20 wt% at different immersion time are reported (Figure 7).

The peak at around 3675 cm^−1^ that is observed at T0 and T7 and disappears at a higher immersion time is related to the -OH stretching vibration of Mg NPs, indicating the presence of hydroxyl group on the surface of low-coordination sites [36]. Furthermore, it is possible to observe the peak associated to the physically adsorbed water molecules at 3450 cm^−1^ [37]. Additional peaks at approximately 2950 (asymmetric) and 2864 cm^−1^ (symmetric) are related to the stretching CH_2_ bond, while the signal at 1460 cm^−1^ corresponds to the CH_2_ bending vibration [38]. The characteristic peak of the PCL component is located at 1725 cm^−1^, which belongs to carbonyl stretching [39].

The bands at 1294 cm^−1^ and 1167 cm^−1^ are assigned to the C-C and C-O stretching vibrations of the crystalline and amorphous phase, respectively [40]. On the other hand, the peaks at 1173 cm^−1^ and 1040 cm^−1^ can be related to symmetric COC stretching and COH stretching, respectively [41]. Aside from these characteristic peaks, two additional peaks appeared with the electrospun nanofibers degradation: one broad peak at around 3500 cm^−1^ due to the presence of O-H (hydroxyl) group, and another one at 1640 cm^−1^ related to the carboxyl group (COOH) due to the hydrolysis of ester bonds [32].

Moreover, the crystallinity index (CI%) was calculated as the ratio between the absorbance peak at 1294 and 1167 cm^−1^ [40], and these were compared with the values obtained by the XRD analysis, which was calculated as reported in our previous work [29]. This comparison is shown in Table 1.

**Figure 8:**
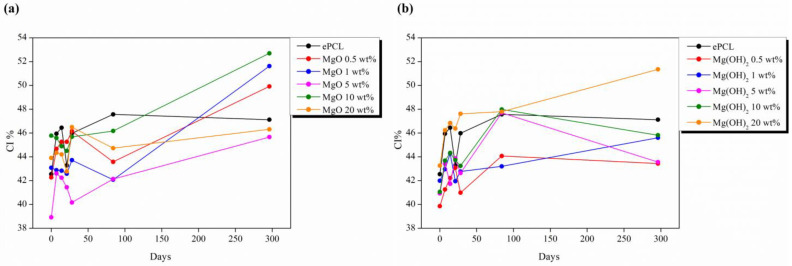
CI % with time changing of (**a**) ePCL + MgO and (**b**) ePCL + Mg(OH)_2_.

The CI% obtained by FTIR are in agreement with those obtained by the XRD analysis. Furthermore, all the samples showed a very high CI% that increased after 296 days with respect to the values calculated at T0. In particular, this phenomenon was observed in the samples with the highest number of NPs, which presented the lowest CI% at T0. Figure 8 reports the variation of CI% with the time change calculated by the FTIR analysis.

**Figure 9:**
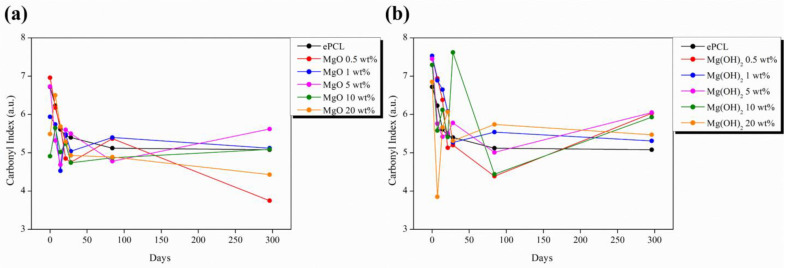
Carbonyl index of (**a**) ePCL + MgO NPs and (**b**) ePCL + Mg(OH)_2_ NPs.

These results indicate that degradation first involves the polymeric chains in the amorphous phase, leading to an increase in crystallinity [32]. In line with this, it is reported that the in vitro degradation occurs in two steps. First, the water absorption causes the degradation of the amorphous zone. The diffusion of water is facilitated in the less organized regions [42]. Then, when the amorphous regions are almost totally degraded, degradation reaches the crystalline phase starting from the surface and moving towards the center [43]. In order to analyze the degradation of the electrospun nanofibers, the carbonyl index was calculated, which is the ratio between the absorbance of carbonyl at 1725 cm^−1^ and of CH_2_ at 1460 cm^−1^ [44]; the results are shown in Figure 9.

In all the samples, except for ePCL + MgO 10 wt%, a reduction in the carbonyl index was observed, with degradation from T0 to T296. However, in general, a reduction in the carbonyl index of 20–30% was observed, with a maximum of 46% for ePCL + MgO 1 wt%. The reduction in the carbonyl index, which is caused by the chain cleavage that occurs through carbonyl linkage, demonstrated the occurring degradation of the electrospun nanofibers.

**Figure 10:**
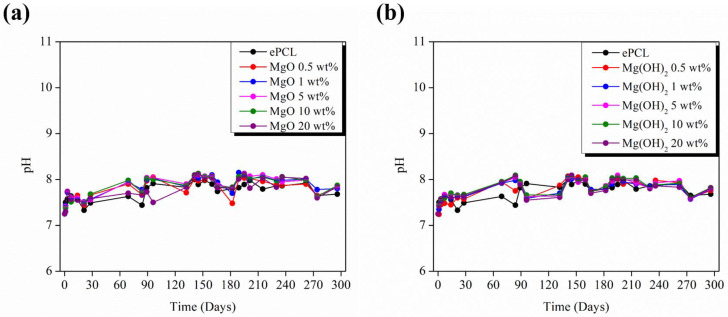
Variation of pH with time (**a**) ePCL + MgO NPs, (**b**) ePCL + Mg(OH)_2_ NPs.

Moreover, considering that PCL degradation consists in the ester bond breakage and the release of its degradation products containing acidic carboxyl groups, which causes the acidification of the medium, it is important to study the pH variation during degradation (Figure 10). A different behavior was observed between the pure matrix and ePCL reinforced with NPs; the pH of ePCL remained almost constant around the value of 7.4 throughout the entire experiment. ePCL with both NPs in all concentrations showed a different behavior; the pH increased, with values above 8 reaching a maximum of 8.13.

This rise in pH can be related to the Mg ions release that induce the alkalinity of the medium and compensate the effect of the acidic degradation byproducts with a neutralizing effect. Such neutralizing behavior of the reinforcement can prevent possible inflammation caused by the implantation of any possible medical device due to acidic degradation byproducts [24,45]. In fact, the first increase was followed by a gradual reduction in pH until the initial values.

After studying the various effects of the in vitro degradation of the electrospun nanofibers in terms of morphological changes, crystallinity and degradation rate, the degradation in SBF was performed to study the bioactivity of the fabricated materials by allowing the growth of calcium phosphate apatites and, in particular, of hydroxyapatite.

**Figure 11:**
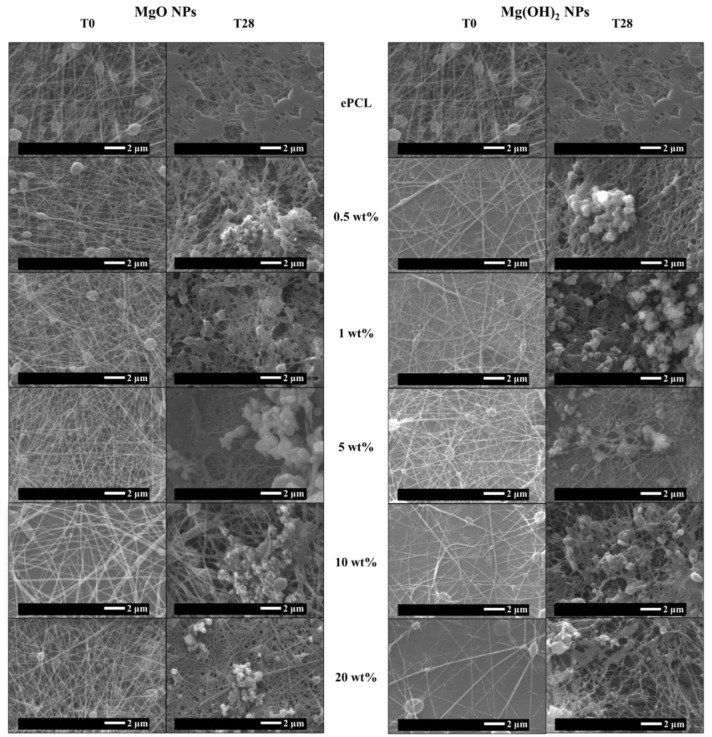
SEM images of ePCL + MgO and Mg(OH)_2_ at T0 and after 28 days of immersion in SBF.

Therefore, the morphology of the electrospun nanofibers was studied by SEM, as reported in Figure 11, which represents a comparison of the electrospun fibers’ morphology after 28 days with the electrospun nanofibers at T0 for the different systems studied. It is possible to see that the electrospun nanofibers lost their morphology. Moreover, in the case of ePCL nanofibers, the surface became rougher, whereas in the electrospun nanofibers reinforced with both types of NPs the presence of precipitated salts was also observed, which can be related to a mineralization process and the precipitation of apatites, confirming our previous results.

**Figure 12:**
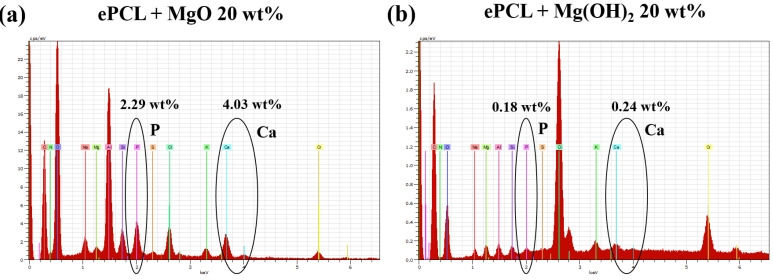
EDX analysis of (**a**) ePCL + MgO 20 wt% and (**b**) ePCL + Mg(OH)_2_. In the circles the signals for P and Ca are shown.

Once the morphology of the electrospun nanofibers had been studied, the materials were characterized by an EDX analysis. In order to determine the presence of hydroxyapatite, the calcium/phosphate (Ca/P) ratios were calculated and summarized in Table 2. Only the systems reinforced with 20 wt% of NPs showed the highest values of the Ca/P ratio; therefore, as an example, the EDX analysis of nanofibers with 20 wt% of NPs is reported in Figure 12.

In particular, the samples that showed Ca/P ratio values around 0.5 can be related to the formation of monocalcium phosphate with the chemical formula Ca(H_2_PO_4_)_2_ [46].

ePCL+ MgO 10 wt% presented a Ca/P ratio of 1.17, which can be reconducted to the formation of two possible products, namely dicalcium phosphate or Brushite with the chemical formula CaHPO_4_ and the β-form of tricalcium phosphate Ca_3_(PO_4_)_2_ [47,48].

ePCL+ MgO 20 wt% and Mg(OH)_2_ 20 wt% presented the highest values of Ca/P ratio of 1.76 and 1.33, respectively, which are closer to that of HA (1.67) [49].

Furthermore, the EDX analysis showed the presence of other elements in the nanofibers, such as Na, Cl, Si, Mg and K.

**Figure 13:**
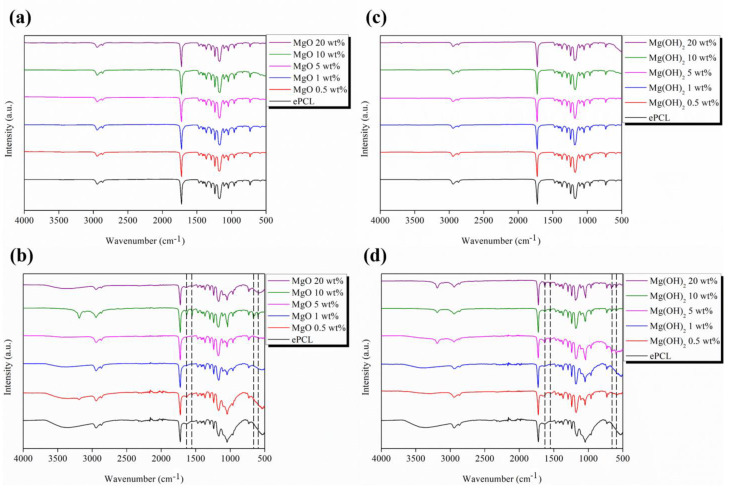
IR spectra of (**a**) ePCL + MgO at T0, (**b**) ePCL + MgO at T28 and (**c**) ePCL +Mg(OH)_2_ at T0. (**d**) ePCL +Mg(OH)_2_ at T28.

Additionally, in order to better analyze these systems, Figure 13 reports the IR spectra of ePCL and ePCL reinforced with MgO and Mg(OH)_2_ NPs at T0 and T28.

It is possible to observe some differences between the spectra at T0 and T28 reinforced with both NPs. In fact, all the samples showed new signals due to the degradation process: one broad peak at 3400 cm^−1^ related to the -OH stretching vibrations, and another signal around 1630 cm^−1^ that can be attributed to the carboxyl groups formed after the degradation [32]. Furthermore, after 28 days, the signals related to the growth of calcium apatite can be seen: in particular, two new sharp peaks at 596 cm^−1^ and 660 cm^−1^ that can be attributed to the bending vibrations of PO_4_^3−^ and another signal at 909 cm^−1^ of the HPO_4_^2−^ group [48]. Another relevant signal appeared at 1628 cm^−1^ that is attributed to hydroxyl liberation mode, and another peak at 1550 cm^−1^ is indicative of the vibrations of CO_3_^2−^ [50,51]. As expected, no signals related to any apatite were observed in the ePCL spectrum.

**Figure 14:**
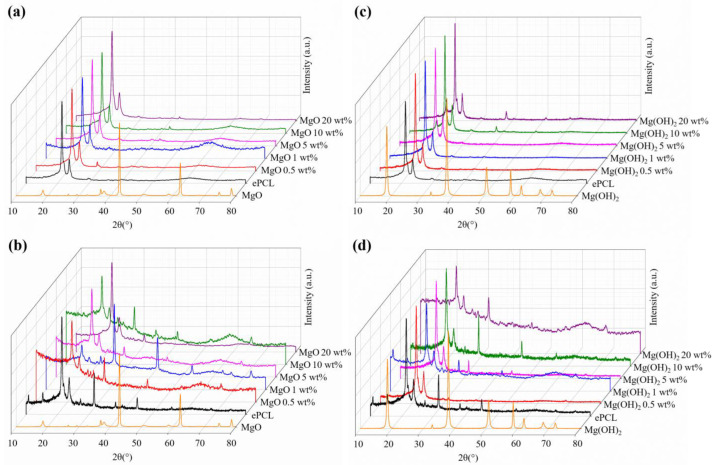
XRD patterns of (**a**) ePCL + MgO NPs at T0, (**b**) ePCL + MgO NPs at T28, (**c**) ePCL + Mg(OH)_2_ NPs at T0 and (**d**) ePCL + Mg(OH)_2_ NPs at T28 in SBF.

Finally, Figure 14 shows the XRD diffractograms of ePCL-based nanofibers before and after 28 days of immersion.

The peaks related to PCL appear at 2θ = 21.5° and 23.9°, which are attributed to the crystallographic planes [110] and [200]. For PCL + NPs at T0, it is possible to observe the diffraction pattern at 2θ = 42.9° and 62.3° attributed to the crystallographic planes (200) and (220) of MgO NPs, and the peaks at 2θ = 18.6°, 38.0°, 50.9° and 58.7° can be ascribed to the lattice planes (001), (101), (102) and (110) of Mg(OH)_2_ [29], which disappeared at T28. Furthermore, the signals related to precipitated salts, such as NaCl and KCl, can be observed at 2θ = 27.3°, 45.5° and 56.3° of NaCl, and at 2θ = 28.3°, 53.7° of KCl, while the signals at 2θ = 26.4° and 31.7° can be attributed to the presence of apatites [50,52].

## 3. Materials and Methods

Poly(ɛ-caprolactone) (PCL) (PCL CAPA 6500, Mn 50 000 g mol^−1^, 0.5 wt%—caprolactone monomer) was supplied by Perstorp® Sweden.

Chloroform (CHCl_3_) (99.6% purity) and N,N-dimethylformamide (DMF) (99.5% purity) from Sigma-Aldrich were used as solvents.

Two types of nanoparticles were used as reinforcement: magnesium oxide nanoparticles (MgO, purity: 99.9%, APS: 20 nm) and magnesium hydroxide nanoparticles (Mg(OH)_2_, purity: 99%, APS:10 nm), which were supplied by Nanoshel.

The electrospun nanofibers were prepared using an Electrospinner Y-flow 2.2.D-XXX (Nanotechnology Solutions) in vertical standard configuration coupled to coaxial concentric needles, following the protocol that we used in our previous work [29].

### 3.1. Degradation Study and Bioactivity

The in vitro degradation study was carried out by immersing samples of 1 cm^2^ of each PCL-based electrospun fiber mat in 20 mL of phosphate buffered saline solution (PBS) and maintained at 37 °C in an oven throughout the entire experiment.

The PBS medium was renovated each week, and the pH was measured as well in order to evaluate any possible variation with a pH METER-02 (Homtiky) with an error of ± 0.01.

The degradation process was studied at different times, named T_X_, where x represents the immersion time, at 1, 3, 7, 14, 21, 28, 84 and 296 days, which is considered the last day of the experiment, while T0 was considered as reference. The samples were dried for 14 days in a vacuum chamber and characterized at each extraction time.

The bioactivity of the fabricated materials was studied by immersing samples of 1 cm^2^ of each PCL-based electrospun fiber mat in 20 mL of simulated body fluid (SBF), which was prepared according to the protocol reported by Kokubo et al. [53] and renovated/changed every 7 days.

The samples were maintained at 37 °C in a UNITRONIC-ORBITAL shaking precision thermostatic bath. The samples were taken after 1, 7, 14, 21, 28 and 112 days (after 4 months) and dried under vacuum for 14 days before testing.

### 3.2. Characterization Techniques

The morphological study of the electrospun fibers was studied by scanning electron microscopy (SEM) (PHILIPS XL30 Scanning Electron Microscope).

All the samples were previously gold-coated (≈5 nm thickness) in a Polaron SC7640 Auto/Manual Sputter.

The SEM images were analyzed with ImageJ software and diameters were calculated as the average value of 30 random measurements for each sample.

X-ray diffraction measurements were carried out using a Bruker D8 Advance instrument with a CuK as source (0.154 nm) and a Detector Vantec1. The scanning range was 2°and 80°, and the step-size and count time per step were 0.023851° and 0.5 s, respectively.

Attenuated total reflectance-Fourier transform infrared spectroscopy (ATR-FTIR) measurements were performed by a Spectrum One FTIR spectrometer (Perkin Elmer instruments). Spectra were obtained in the range of 4000–400 cm^−1^ with a resolution of 4 cm^−1^.

Finally, an energy-dispersive X-ray spectroscopy (EDX) analysis was conducted using Hitachi SU 8000 FE-SEM equipment with a Bruker XFlash Detector 5030 working at 15 kV.

## 4. Conclusions

In this work, after the obtention of PCL-based electrospun nanofibers reinforced with MgO and Mg(OH)_2_, we focused our attention on the study of hydrolytic degradation in PBS and bioactivity in SBF of the fabricated materials. The degradation of the materials took almost a year, as expected for this polymer, which is characterized by a slow degradation rate. However, the average diameter for ePCL remained almost constant throughout the entire experiment, with a slight decrease after 296 days, while nano-reinforced nanofibers showed a higher decrease: in particular, with 20 wt% of NPs with a reduction in diameter of 43% and 46% for ePCL + MgO 20 wt% and ePCL + Mg(OH)_2_ 20 wt%, respectively. From a morphological point of view, we observed the fibers rupture after 84 days and witnessed the precipitation of minerals on the fibers surface, which was also confirmed by an XRD analysis. The crystallinity of the materials, which was calculated from an XRD and FTIR analysis, increased with respect to the initial values, proving that degradation first involves the amorphous region, leading to an increase in crystallinity. Furthermore, an IR analysis showed the variation of the signals related to the ester bond and degradation byproduct. Moreover, the calculation of the carbonyl index, which is directly related to the intensity of the carbonyl (ester) signals, is a better parameter to evaluate the degradation process; we observed a reduction in the carbonyl index of around 20–30%, and of almost 50% for ePCL + MgO 1 wt% after 296 days. Finally, pH showed similar values throughout the entire experiment, assuming values of around 7.6; however, in the case of nanofibers reinforced with NPs, we observed an initial rise in pH due to the release of Mg ions, followed by a subsequent reduction.

To conclude, even if the degradation of the materials takes more than 3 months, the presence of NPs seems to have an accelerating effect on the degradation of the electrospun nanofibers based on PCL. Additionally, the bioactivity of PCL-based electrospun nanofibers was studied, as well as the formation of crystals on the nanofibers surface, except for neat ePCL—in particular, related to the formation of NaCl and apatites—which we also confirmed using FTIR can be related with the signals related to the phosphate and carbonate group. Moreover, from the XRD analysis, the signals observed at 2θ = 26.4° and 31.7° can be attributed to the presence of apatites. Finally, an EDX analysis was carried out in order to confirm the presence of Ca and P and calculate the Ca/P ratio. Only the samples with 20 wt% of NPs presented the highest Ca/P values, which agree well with the value reported for natural HA.

## Figures and Tables

**Table 1:** CI% values calculated by XRD and FTIR at T0 and T296 for the different systems.

	XRD		FTIR	
Sample	T0	T296	T0	T296
ePCL	53	50	43	47
ePCL+ MgO 0.5 wt%	52	52	42	50
ePCL+ MgO 1 wt%	52	51	43	52
ePCL+ MgO 5 wt%	52	57	39	46
ePCL+ MgO 10 wt%	49	54	46	53
ePCL+ MgO 20 wt%	48	57	44	46
ePCL+ Mg(OH)_2_ 0.5 wt%	51	43	40	43
ePCL+ Mg(OH)_2_ 1 wt%	50	49	42	46
ePCL+ Mg(OH)_2_ 5 wt%	48	44	41	44
ePCL+ Mg(OH)_2_ 10 wt%	46	52	41	46
ePCL+ Mg(OH)_2_ 20 wt%	37	51	43	51

**Table 2:** Calculated Ca/P ratio values of ePCL, ePCL + MgO NPs and ePCL + Mg(OH)_2_ NPs at T0 and after 28 days of immersion in SBF.

	T0	T28
ePCL	-	0.70
ePCL+ MgO 0.5 wt%	-	0.18
ePCL+ MgO 1 wt%	-	0.19
ePCL+ MgO 5 wt%	-	0.65
ePCL+ MgO 10 wt%	-	1.17
ePCL+ MgO 20 wt%	-	1.76
ePCL+ Mg(OH)_2_ 0.5 wt%	-	0.27
ePCL+ Mg(OH)_2_ 1 wt%	-	0.56
ePCL+ Mg(OH)_2_ 5 wt%	-	0.53
ePCL+ Mg(OH)_2_ 10 wt%	-	0.43
ePCL+ Mg(OH)_2_ 20 wt%	-	1.33

## Data Availability

Not applicable.
